# Mr. Davies, on Rheumatism

**Published:** 1806-12

**Authors:** H. Davies

**Affiliations:** Piccadilly


### *To the Editors of the Medical and Phyſical Journal.*

Gentlemen,

RHEUMATISM, in this our varying clime, is a disease which very generally prevails, and proves a source of considerable pain to the patient, as well as perplexity to the practitioner; therefore any hint, respecting an improvement in the treatment of this afflictive disorder, will not, it is hoped, be deemed an obtrusion on a page of your useful miscellany.

Rheumatism has been very properly distinguished by the terms Acute and Chronic; but, as the treatment of the former has been, for the most part, uniformly the same, by adopting the antiphlogistic plan, I shall forbear making any remark, and confine my observations solely to the management of the latter.

The late eminent Dr. Cullen, according to his accustomed perspicuity, has defined chronic rheumatism, as to its proximate cause, to be an "atony of both the blood vessels vessels, and of the muscular fibres of the part affected, together with a degree of rigidity and contraction in the latter, such as frequently attends them in a state of atony;" and he very properly adds, that the general indication of cure must be, "to restore the activity and vigour of the vital principle in the part." Hence guaiacum, volatile alkaline salts, essential oil, &c. have been adopted as remedies, but, perhaps, not with that success which has been ardently wished for.

I have of late seen the most beneficial effects resulting from the free exhibition of *opium*, accompanied with the cinchona, in this troublesome complaint. From gr. 1 fs. to gr. ij. of the former, administered 5t a vel 6t à quaq. hor. with a draught of the decoct. cinchon. rendered grateful by the addition of a little aromatic tincture, has alleviated the excruciating pains attached to this disorder, induced a perspirable state of the system, and by persevering in the same plan for a little time, entirely liberated the patient from its tormenting influence.

In addition to this *internal* mode of treatment, a liniment, composed of equal parts of the linim. camphor. & sp. tereb. rect. should be well applied to the parts affected, night and morning; covering them afterwards with a piece of new flannel, or fleecy hosiery.

The pleasing warmth excited by this stimulating application, affords considerable relief, and, perhaps, the boasted effects attributed to the celebrated essence of mustard, are owing to the terebinthinate quality, which it is well known to possess.

I do not by any means pretend to a new discovery by this mode of treatment in the chronic rheumatism, but rather urge a plan, the good effects of which I have satisfactorily witnessed in practice; and should the adoption of it prove successful, under the management of other practitioners, the end of conveying this hint will be fully answered; whilst it will afford a pleasure in having an opportunity of contributing a mite to the treasury of medical improvement.

I am, &c.

H. DAVIES.

*Piccadilly, Oct. 9, 1806.*

